# Guideline recommendations on minimal blood vessel diameters and arteriovenous fistula outcomes

**DOI:** 10.1177/11297298231180627

**Published:** 2023-06-19

**Authors:** Letty V van Vliet, Niek Zonnebeld, Jan H Tordoir, Wouter Huberts, Lee H Bouwman, Philippe W Cuypers, Stefan G Heinen, Laurens C Huisman, Susan Lemson, Barend ME Mees, Felix J Schlösser, André A de Smet, Raechel J Toorop, Tammo Delhaas, Maarten G Snoeijs

**Affiliations:** 1 Department of Biomedical Engineering, CARIM School for Cardiovascular Diseases, Maastricht University, Maastricht, the Netherlands; 2 Department of Vascular Surgery, Maastricht University Medical Centre, Maastricht, the Netherlands; 3 Department of Surgery, Zuyderland Medical Centre, Heerlen, the Netherlands; 4 Department of Surgery, Catharina Hospital, Eindhoven, the Netherlands; 5 Department of Surgery, St. Antonius Hospital, Nieuwegein, the Netherlands; 6 Department of Surgery, Flevoziekenhuis, Almere, the Netherlands; 7 Department of Surgery, Slingeland Hospital, Doetinchem, the Netherlands; 8 Department of Surgery, Laurentius Hospital, Roermond, the Netherlands; 9 Department of Surgery, Maasstad Hospital, Rotterdam, the Netherlands; 10 Department of Surgery, University Medical Centre Utrecht, Utrecht, the Netherlands

**Keywords:** Arteriovenous fistula, hemodialysis, preoperative diameter, vascular access function, cohort study

## Abstract

**Objective:**

Clinical guidelines provide recommendations on the minimal blood vessel diameters required for arteriovenous fistula creation but the evidence for these recommendations is limited. We compared vascular access outcomes of fistulas created in agreement with the ESVS Clinical Practice Guidelines (i.e. arteries and veins >2 mm for forearm fistulas and >3 mm for upper arm fistulas) with fistulas created outside these recommendations.

**Methods:**

The multicenter Shunt Simulation Study cohort contains 211 hemodialysis patients who received a first radiocephalic, brachiocephalic, or brachiobasilic fistula before publication of the ESVS Clinical Practice Guidelines. All patients had preoperative duplex ultrasound measurements according to a standardized protocol. Outcomes included duplex ultrasound findings at 6 weeks after surgery, vascular access function, and intervention rates until 1 year after surgery.

**Results:**

In 55% of patients, fistulas were created in agreement with the ESVS Clinical Practice Guidelines recommendations on minimal blood vessel diameters. Concordance with the guideline recommendations was more frequent for forearm fistulas than for upper arm fistulas (65% vs 46%, *p* = 0.01). In the entire cohort, agreement with the guideline recommendations was not associated with an increased proportion of functional vascular accesses (70% vs 66% for fistulas created within and outside guideline recommendations, respectively; *p* = 0.61) or with decreased access-related intervention rates (1.45 vs 1.68 per patient-year, *p* = 0.20). In forearm fistulas, however, only 52% of arteriovenous fistulas created outside these recommendations developed into a timely functional vascular access.

**Conclusions:**

Whereas upper arm arteriovenous fistulas with preoperative blood vessel diameters <3 mm had similar vascular access function as fistulas created with larger blood vessels, forearm arteriovenous fistulas with preoperative blood vessel diameters <2 mm had poor clinical outcomes. These results support that clinical decision-making should be guided by an individual approach.

## Introduction

Non-maturation of newly created arteriovenous fistulas is an important clinical problem leading to additional interventions and prolonged central venous catheter dependence in patients starting hemodialysis treatment.^1,2^ Therefore, the preoperative assessment of these patients is focused on finding a combination of good quality arteries and veins that has a high likelihood of developing into a functional vascular access. Historically, the diameter of these blood vessels has been regarded as one of the primary indicators of the suitability for a native arteriovenous fistula. Ultrasound allows precise measurement of blood vessel diameters before fistula creation and this has been shown to improve vascular access outcomes as compared to preoperative physical examination alone.^3,4^ However, the minimal blood vessel diameters that are considered acceptable for creation of these fistulas vary between dialysis units. Even international clinical practice guidelines on vascular access provide different recommendations: the European Society for Vascular Surgery (ESVS) advises minimal arterial and venous diameters of 2 mm in the forearm and 3 mm in the upper arm,^5^ whereas the KDOQI 2019 Update provides a weak recommendation to use blood vessels smaller than 2 mm only after critical assessment of their quality.^6^

Just before the ESVS Clinical Practice Guidelines on vascular access were published, we performed the prospective multicenter Shunt Simulation Study, resulting in a representative cohort of patients with end-stage renal disease receiving their first arteriovenous fistula with detailed information on preoperative blood vessel characteristics and vascular access function.^7^ We noticed that many patients received arteriovenous fistulas using arteries and veins smaller than recommended by the ESVS Clinical Practice Guidelines published in June 2018. Since all patients enrolled in the study were operated on before this time, there could be no bias resulting from deliberate deviation from published guidelines by our vascular surgeons. The Shunt Simulation Study cohort therefore provides an excellent opportunity to investigate the validity of these recommendations by analyzing its arteriovenous fistula outcomes according to concordance with the guidelines.

## Methods

### Shunt Simulation Study

The Shunt Simulation Study was a multicenter randomized controlled trial that evaluated the clinical impact of a personalized computational model to predict postoperative fistula flow.^7^ The trial was approved by the institutional review board azM/UM and was registered at ClinicalTrials.gov (NCT02453412). In nine Dutch hospitals, consenting patients who required creation of a new arteriovenous fistula were enrolled in the trial. All patients had preoperative physical examination and duplex ultrasound measurements. In the intervention group, surgeons received additional blood flow predictions to help them decide on the most appropriate arteriovenous fistula configuration. All patients were followed for 1 year after access creation, unless they died, received a kidney transplantation, or withdrew from the study. During follow-up, patients received duplex measurements at 6 weeks, 6 months, and 1 year after vascular access creation, and clinical outcomes were recorded.

### Study design

In this secondary analysis of the Shunt Simulation Study, we compared outcomes of patients with fistulas created in agreement with the ESVS Clinical Practice Guidelines on minimal blood vessel diameters to outcomes of patients with fistulas created outside these recommendations. We considered fistulas as having been created outside guideline recommendations when the smallest segment of the artery and/or the smallest segment of the vein of the arteriovenous fistula was smaller than recommended (i.e. at least 2 mm for forearm fistulas and at least 3 mm for upper arm fistulas). Patients were excluded from the analysis when they did not receive a radiocephalic, brachiocephalic, or brachiobasilic arteriovenous fistula. Since the recommendations on minimal blood vessel diameters in the ESVS Clinical Practice Guidelines are different for forearm and upper arm fistulas, we analyzed outcomes in these subgroups separately.

### Preoperative duplex ultrasound

Preoperative duplex ultrasound measurements were performed according to the Shunt Simulation Study protocol by a trained vascular technician in a warm room (>*20°C*), with the patient in supine position.^8^ Upper extremity arteries and veins were scanned for continuity and local diameter reductions. Blood vessel diameters were measured from inner wall to inner wall at three different sites for each artery and vein (proximal, mid, and distal). The smallest of the three diameters was registered as the blood vessel diameter for the analyses in this study. Venous diameters were measured in the transverse plane using a tourniquet, whereas arterial diameters were measured in the longitudinal plane. Venous diameters were calculated as the mean of the antero-posterior and lateral diameters.

### Vascular access outcomes

Maturation assessed by duplex ultrasound measurements was defined as a flow of ⩾500 mL/min and a vein diameter of ⩾4 mm at 6 weeks after surgery.^9^ Vascular access function was defined differently for patients on dialysis at the time of access creation and for predialysis patients. For patients on dialysis, the time to a functional fistula was defined according to the ESVS Clinical Practice Guidelines as a fistula that was cannulated with two needles for at least six hemodialysis sessions at the prescribed access circuit flow within 30 days. In predialysis patients, we considered the vascular access to be functional when the index fistula could be used at dialysis initiation without first needing a central venous catheter.^5^ Predialysis patients who did not start dialysis treatment within the study period were excluded from this analysis. All access-related interventions after vascular access creation—including surgical, endovascular, and central venous catheter interventions—were registered to calculate the intervention rate per patient-year. Access-related interventions done before the fistula was functional were counted as interventions for maturation, and interventions done later were counted as interventions for maintenance of the arteriovenous fistula.

### Statistical methods

Categorical variables are reported as percentages and differences between groups were tested using Chi square tests and Fisher’s exact tests. Continuous variables are shown as means ± standard deviations when normally distributed or as medians (interquartile range) when not normally distributed. Student’s *t*-test was used to test differences between groups for normally distributed continuous variables; the Mann-Whitney *U*-test was used for continuous variables that were not normally distributed. Intervention rates were calculated by dividing the number of interventions by the patient-time in the study groups. Intervention rates were compared using Poisson distribution and test-based methods to construct confidence intervals. In patients on dialysis, the proportion of functional fistulas over time was analyzed with Kaplan-Meier estimates and compared with log-rank tests. Multivariable logistic regression models were made to predict clinical outcomes. Predictor variables added to the regression models included compliance to the guideline recommendations on minimal blood vessel diameters as well as the following clinically relevant predictors of vascular access function: age, sex, body mass index, diabetes, and fistula configuration. The assumptions of linearity, multicollinearity, and independence of errors were checked. *p* Values <0.05 were considered statistically significant. Statistical analysis was done with IBM SPSS Statistics for Windows, version 27 (IBM Corp., Armonk, NY).

## Results

### Study population

**Figure 1.**
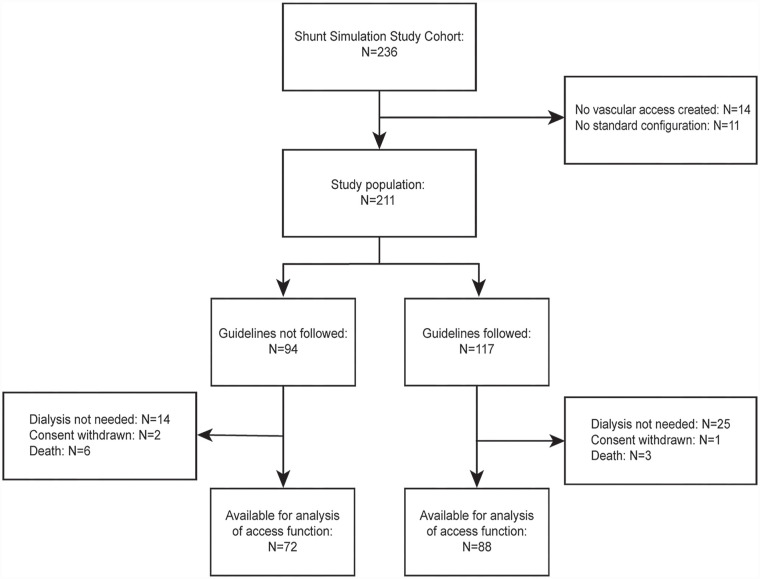
Flow chart. The study population was divided according to concordance with the ESVS Clinical Practice Guidelines on minimal blood vessel diameters for arteriovenous fistula creation.

From June 2015 until March 2018, 236 patients were enrolled in the Shunt Simulation Study (Figure 1). In 14 patients no vascular access was created and 11 patients received no standard configuration, resulting in a study population of 211 patients for the current analysis. In 94 patients (45%), the arteriovenous fistulas created were outside the ESVS guideline recommendations on minimal blood vessel diameters. The postoperative flow predictions offered as part of the Shunt Simulation Study did not influence agreement with the clinical guideline (53% agreement in patients with flow predictions vs 58% in patients in the control group, *p* = 0.41). In total, 160 patients (76%) were available for the analysis of vascular access function at 1 year. The most common reason for a patient not being available for the analysis of vascular access function was not needing dialysis treatment (*N* = 39), followed by death (*N* = 9) and withdrawal of consent (*N* = 3).

### Baseline characteristics

Baseline characteristics of the two patient groups are shown in Table 1. Age, sex, and comorbidities were similar in both study groups. Patients with fistulas created outside guideline recommendations had a significantly lower body mass index compared to patients with fistulas created in agreement with the guidelines (27 vs 29 kg/m^2^, *p* = 0.02). Concordance with the guideline recommendations was more frequent for forearm fistulas than for upper arm fistulas (65% vs 46%, *p* = 0.01). Although there were less radiocephalic fistulas in patients in whom the guidelines had not been followed, preoperative diameters of the veins used in these patients were still smaller than in patients with fistulas created in agreement with the guidelines (2.2 vs 3.2 mm, *p* < 0.01).

**Table 1.** Baseline characteristics.

	Fistulas created in agreement with guideline recommendations		*p*
	No	Yes	
	*N* = 94	*N* = 117	
Age (years)	66 (14)	65 (11)	0.82
Sex (male)	59%	71%	0.06
Body mass index (kg/m^2^)	27 (6)	29 (6)	0.02
*Comorbidities*			
Diabetes	45%	39%	0.43
Hypertension	83%	88%	0.30
On dialysis	41%	41%	0.95
*Duplex measurements*			
*Arterial diameter (mm)*			
All patients	3.1 (1.0)	3.2 (0.9)	0.50
Radiocephalic fistulas	2.0 (0.3)	2.5 (0.4)	<0.01
Brachiocephalic fistulas	3.7 (0.7)	4.1 (0.7)	0.02
Brachiobasilic fistulas	3.6 (0.4)	3.9 (0.6)	0.23
* Venous diameter (mm)*			
All patients	2.2 (0.6)	3.2 (0.9)	<0.01
Radiocephalic	1.8 (0.5)	2.7 (0.6)	<0.01
Brachiocephalic	2.4 (0.5)	3.7 (0.5)	<0.01
Brachiobasilic	2.6 (0.3)	4.2 (0.9)	<0.01
*Arteriovenous fistula configuration*			<0.01
Radiocephalic	37%	56%	
Brachiocephalic	54%	27%	
Brachiobasilic	9%	16%	
*Anesthesia*			0.85
Local	1%	2%	
Regional	68%	65%	
General	31%	33%	

Data are presented as percentages or as mean ± standard deviation.

### Vascular access function

**Figure 2.**
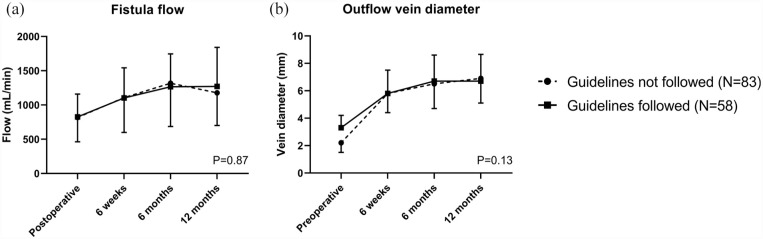
Duplex ultrasound measurements of arteriovenous fistulas created in agreement with guideline recommendations on minimal blood vessel diameters or not. (a) Brachial artery blood flow measured by duplex ultrasound over time; (b) Diameter of the arteriovenous fistula outflow vein measured by duplex ultrasound over time. Data are presented as means and standard deviations. Differences between time points and study groups were analyzed with repeated measures ANOVA. *p* Values refer to comparisons between study groups.

**Figure 3.**
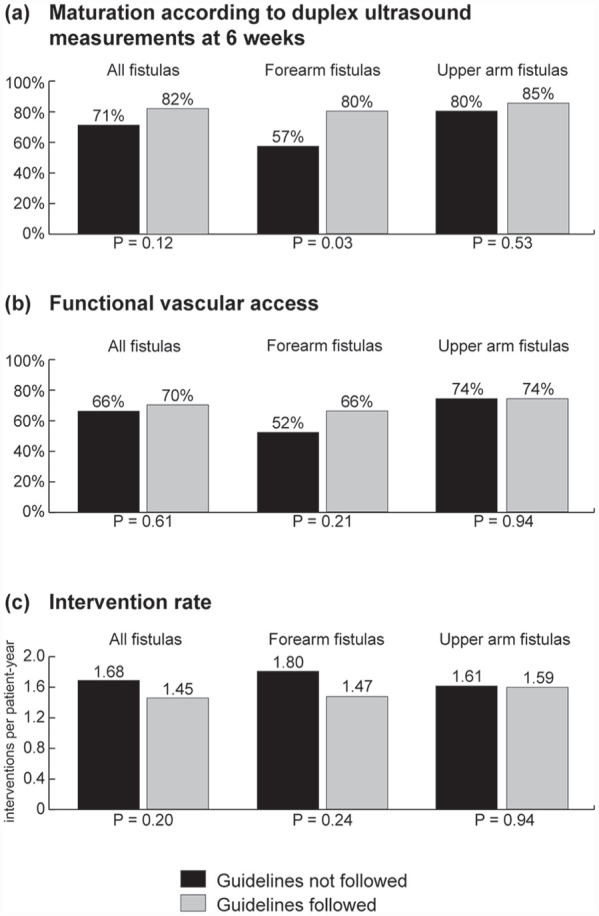
Vascular access function of all fistulas, and separately for forearm and upper arm arteriovenous fistulas created in line with guideline recommendations on minimal blood vessel diameters or not. Bar charts of (a) duplex maturation rate (brachial artery blood flow >500 mL/min and outflow vein diameter >4 mm) determined at 6 weeks after vascular access creation; (b) Vascular access function: for patients on dialysis at the time of vascular access creation or who started dialysis within 4 months after vascular access creation, an optimal vascular access outcome was considered as a functional arteriovenous fistula within 4 months after surgery (cannulation with two needles for at least six hemodialysis sessions at the prescribed access circuit flow in 30 days). For patients who had not yet started dialysis treatment at 4 months after vascular access creation, an optimal vascular access outcome was considered as starting dialysis with the index arteriovenous fistula; (c) Access-related intervention rate. Differences between groups were analyzed with Poisson distribution tests.

Both fistula flow and outflow vein diameter gradually increased over time until 6 months after vascular access creation. The initial difference in pre-operative outflow vein diameter of fistulas created outside the guideline recommendations disappeared at the 6 weeks postoperative measurements. There were no differences in fistula flow and outflow vein diameters between the study groups during the 1-year follow-up (Figure 2). At 6 weeks after surgery, 82% of fistulas created within guideline recommendations had reached maturation according to duplex ultrasound criteria as compared to 71% of the fistulas with smaller blood vessel diameters (Figure 3(a), *p* = 0.12).

**Figure 4.**
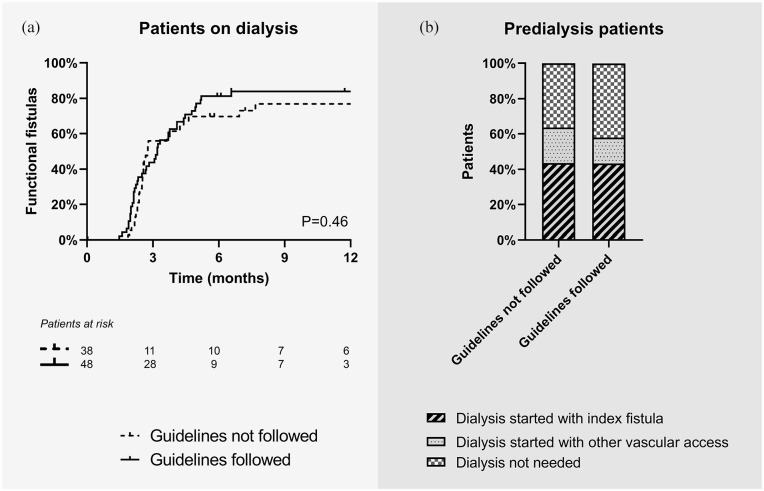
Vascular access function of arteriovenous fistulas created in agreement with guideline recommendations on minimal blood vessel diameters or not. (a) Kaplan-Meier survival curve of time to having a functional fistula (cannulation with two needles for at least six hemodialysis sessions at the prescribed access circuit flow in 30 days). The analysis was restricted to patients on dialysis at the time of vascular access creation. Differences between study groups were analyzed with the log rank test. (b) Bar charts of modality of start of hemodialysis. This analysis was restricted to patients not on dialysis at the time of vascular access creation.

In patients on dialysis at the time of fistula creation, agreement with guideline recommendations on minimal blood vessel diameters did not change the time to functional fistula use (81% for fistulas created within vs 70% for fistulas created outside guideline recommendations at 6 months after surgery, *p* = 0.46; Figure 4(a)). In predialysis patients, initiation of dialysis treatment was similar in the two groups: 44% of both groups started dialysis treatment with the index fistula (Figure 4(b)), whereas 20% of patients with fistulas created outside guideline recommendations started with another vascular access as compared to 14% of patients with fistulas created in agreement with these recommendations. The remaining patients did not need dialysis treatment after 1 year. To combine vascular access outcomes for dialysis and predialysis patients, we defined the “textbook outcome” for vascular access surgery as a fistula that was functional within 4 months for patients who were on dialysis treatment at 4 months after vascular access creation, and as a fistula that was used at dialysis initiation for patients who were not on dialysis treatment at 4 months after vascular access creation. According to this definition, 66% of patients with fistulas created outside guideline recommendations and 70% of patients with fistulas created in agreement with these recommendations had a timely functional vascular access (Figure 3(b)).

Access-related intervention rates in the two study groups were comparable (1.68 interventions per patient-year when fistulas were created outside of the guideline recommendations vs 1.45 interventions per patient-year when fistulas were created in line with these recommendations, Figure 3(c), *p* = 0.20). The rate of interventions for maturation was also similar.

### Predictors of vascular access function

In multivariable regression analysis, concordance with the ESVS guideline recommendations on minimal blood vessel diameters was not associated with improvements in obtaining a timely functional vascular access (OR 0.77, 95% CI: 0.38–1.56) or in decreasing the rate of access-related interventions. Furthermore, age, sex, BMI, and diabetes were neither associated with timely maturation into a functional vascular access nor with increased access-related intervention rates (Table 2). However, patients receiving forearm fistulas had a significantly lower chance of having a functional vascular access within 4 months after surgery (OR 0.50, 95% CI: 0.25–1.00).

**Table 2.** Multivariable logistic regression analysis of optimal vascular access outcome.

	Functional vascular access (within 4 months after creation)*		Intervention rate (⩾2 interventions per patient-year)	
	OR (95% CI)	*p*	OR (95% CI)	*p*
Age (/year)	1.01 (0.98–1.04)	0.56	1.01 (0.99–1.03)	0.47
Sex (male)	1.31 (0.63–2.73)	0.48	1.55 (0.84–2.87)	0.16
Diabetes (yes)	0.95 (0.47–1.93)	0.88	0.97 (0.54–1.73)	0.91
Fistula configuration (forearm)	0.50 (0.25–1.00)	0.05	1.15 (0.65–2.06)	0.63
Fistulas created in agreement with guideline recommendations (no)	0.77 (0.38–1.56)	0.47	1.58 (0.88–2.85)	0.13

* For patients on dialysis at the time of vascular access creation or who started dialysis within 4 months after vascular access creation, an optimal vascular access outcome was considered as a functional arteriovenous fistula within 4 months after surgery (cannulation with two needles for at least six hemodialysis sessions at the prescribed access circuit flow in 30 days). For patients who had not yet started dialysis treatment at 4 months after vascular access creation, an optimal vascular access outcome was considered as starting dialysis with the index arteriovenous fistula.

### Vascular access function in subgroups of forearm and upper arm fistulas

Subgroup analysis showed that forearm fistulas created outside the ESVS guideline recommendations on minimal blood vessel diameters had a significantly lower duplex maturation rate at 6 weeks after surgery compared to all other fistulas (57% vs 81%, *p* < 0.01). Furthermore, only 52% of forearm fistulas created outside the guideline recommendations developed into a timely functional vascular access as compared to 72% of all other fistulas (*p* = 0.02). The poor functional outcomes of these forearm fistulas appear to drive the non-statistically significant differences observed between the full study groups. The access-related intervention rates did not differ significantly between forearm fistulas created outside the guideline recommendations and the other fistulas (1.80 vs 1.55 interventions per patient-year, *p* = 0.32).

Further sensitivity analysis showed that vascular access outcomes did not change with the number of sites (proximal, mid, and distal) at which the vessel diameter was below the recommended cut-off value. Furthermore, there was no difference in vascular access outcomes when comparing patients with fistulas discordant for the arterial, the venous, or both diameter cut-offs.

## Discussion

The ESVS Clinical Practice Guidelines recommend minimal arterial and venous diameters of 2 mm for creation of forearm fistulas and minimal diameters of 3 mm for creation of upper arm fistulas.^5^ In this observational multicenter study of arteriovenous fistulas created before publication of these guidelines, only 55% of the fistulas were created in line with its recommendations. As fistulas matured after surgery, the smaller veins of fistulas created outside the guideline recommendations increased in size and became as large as the fistulas created in agreement with the guidelines. Overall, concordance with the guideline recommendations was neither associated with an increase in the proportion of functional fistulas, nor with a decrease in the intervention rate to achieve or maintain function. However, forearm fistulas with blood vessel diameters smaller than 2 mm formed a subgroup of vascular accesses with poor function, with only 52% of these fistulas becoming functional.

Since the findings of our study seem to be at odds with the ESVS Clinical Practice Guidelines, it is of interest to examine the scientific evidence underpinning their recommendations. Ideally, clinical recommendations on the minimal blood vessel diameters required for creation of an arteriovenous fistula should be based on studies comparing cohorts in which vascular surgeons used different diameter cut-off values. However, neither randomized nor observational comparative studies have been published, and clinical recommendations are therefore based on non-comparative observational studies. These studies generally support a positive correlation between preoperative arterial and venous diameters and postoperative vascular access function.^10,11^ The largest observational study reports on vascular access outcomes of 507 radiocephalic and 237 brachiocephalic fistulas.^12^ These fistulas were created using a local protocol for minimal blood vessel diameters (equal to the ESVS Clinical Practice Guidelines), reducing bias as a result of the subjective assessment of vessel quality by the attending vascular surgeon. Despite this protocol, 7% of radiocephalic fistulas and 28% of brachiocephalic fistulas were created with blood vessel diameters <2 and <3 mm, respectively. From this subset, 58% of radiocephalic fistulas and 80% of brachiocephalic fistulas developed into a functional vascular access. Although the proportion of fistulas created outside the guideline recommendations was much greater in our study, the maturation rate of these fistulas was comparable to fistulas created within guideline recommendations (52% for forearm fistulas <2 mm and 74% for upper arm fistulas <3 mm). Another source of valuable information comes from small observational studies reporting specifically on the outcome of radiocephalic fistulas with small diameters. The proportion of functional fistulas varied widely from 0% with diameters <1.6 mm,^13^ 16% and 42% with diameters <2 mm,^14,15^ and 88% with diameters <2.2 mm.^16^ Taken together, the scientific evidence indicates that although the chance of successful fistula maturation increases with greater blood vessel diameters, there is no strict diameter cut-off that consistently differentiates between fistulas with good and bad clinical outcomes.

Recommending minimal blood vessel diameters for vascular access creation would require that these diameters can be measured accurately. Duplex ultrasound allows precise measurement of blood vessel diameter and its use for surgical planning has been shown to improve vascular access outcomes compared to physical examination alone.^3,4^ Nevertheless, ultrasound-measured blood vessel diameter may vary from day-to-day, from observer to observer, and with different measurement conditions. Studies investigating these conditions found larger vein diameters after warm water immersion,^17^ tourniquet application (resulting in 0.8 mm increase in forearm cephalic vein diameter),^18^ and brachial plexus block (resulting in changes in the surgical plan in 42% of patients).^19^ These techniques are all likely to simulate the venous diameter after distension as a result of the increased blood flow after creation of the fistula. Apart from these measurement conditions, ultrasound assessment of blood vessel diameter may depend on the skills of the operator. Pressure of the ultrasound transducer on the patient’s skin may compress blood vessels and result in different diameter measurements, in particular for superficial veins. In an observational study on 10 patients with end-stage renal disease, we found good reproducibility between observers when measurements were done at the same patient visit (intraclass correlation coefficient: 77%–89%).^20^ However, the day-to-day variability of blood vessel diameter measurements by different observers remains to be determined.

The quality of arteries and veins for vascular access is determined by more factors than their diameters. Vessels of lesser quality may not be capable to accommodate the biological changes of the maturation process and therefore result in worse clinical outcomes. In the Hemodialysis Fistula Maturation study, arterial stiffness, endothelial function, and venous capacitance were measured in 602 patients before vascular access creation.^21^ Arterial stiffness was measured as carotid-femoral and carotid-radial pulse wave velocities and was not associated with postoperative fistula flow and venous outflow diameter. Endothelial function was measured as brachial artery flow-mediated and nitroglycerin-mediated dilation. It follows from Poiseuille’s law that rather small changes in vascular diameter result in large resistance changes. In the study, every 10% increase in brachial artery dilation was associated with 12%–14% greater fistula flow at 6 weeks after surgery. In another observational study on 47 patients with newly created fistulas, brachial artery flow was 200 mL/min lower in patients with pre-existent arterial calcifications at the arteriovenous anastomosis at 6 weeks after surgery.^22^ Finally, in the Hemodialysis Fistula Maturation study venous capacitance was measured using venous occlusion plethysmography and was not associated with vascular access function. However, this technique reflects the capacitance of the entire venous bed of the arm and does not directly measure the distensibility of the vein that will be used for the arteriovenous fistula. When cephalic vein distensibility was measured directly by ultrasound assessment of venous diameters before and after application of a tourniquet in a cohort of 72 patients receiving a radiocephalic fistula, it was a more important predictor of vascular access function than the actual vein diameter.^16^

Imposing strict cut-off values for minimal blood vessel diameters for vascular access creation may result in the elimination of one or more arteriovenous fistula configurations. Reassessment of blood vessel diameters in the operating room may identify additional vascular access opportunities due to the vasodilatory effect of the brachial plexus block.^19^ Furthermore, attempting every possible fistula can be critical in patients with long life expectancies who may eventually run out of vascular access. On the other hand, when arteriovenous fistulas do not become functional because of small blood vessel diameters, additional access-related interventions are required that may affect patient satisfaction and quality of life. To balance these effects, decisions on the blood vessel diameters required for creation of arteriovenous fistulas should be made within the context of individual patients.^23^

In conclusion, upper arm arteriovenous fistulas with preoperative blood vessel diameters <3 mm have similar vascular access function as fistulas created with larger blood vessels, whereas forearm arteriovenous fistulas with preoperative blood vessel diameters <2 mm have poor clinical outcomes. These results support that clinical decision-making should be guided by an individual approach taking into account blood vessel quality, other possibilities for vascular access, the expected time on hemodialysis, and patient preferences instead of by strictly adhering to diameter cut-off values.
